# Dissecting the differential performance of contrasting stay-green and stem reserve mobilization wheat (*Triticum aestivum L.*) genotypes – Validation of GWAS analysis

**DOI:** 10.1371/journal.pone.0339374

**Published:** 2026-01-05

**Authors:** Animireddy China Malakondaiah, Ajay Arora, Hari Krishna, Sukumar Taria, Sudhir Kumar, Sekar Kousalya, Narayana Bhat Devate, Jasdeep Chatrath Padaria, Sahana Police Patil, Pradeep Kumar Singh

**Affiliations:** 1 Division of Plant Physiology, ICAR-Indian Agricultural Research Institute, New Delhi, India; 2 Division of Genetics, ICAR-Indian Agricultural Research Institute, New Delhi, India; 3 ICAR-Central Agroforestry Research Institute, Jhansi, Uttar Pradesh, India; 4 International Center for Agricultural Research in the Dry Areas (ICARDA)-Food Legume Research Platform (FLRP), Amlaha, Madhya Pradesh, India; 5 ICAR-National Institute for Plant Biotechnology, New Delhi, India; Institute of Genetics and Developmental Biology Chinese Academy of Sciences, CHINA

## Abstract

The present study aimed to validate the identified marker trait associations (MTAs) for stay-green (SG) and stem reserve mobilisation (SRM) using 12 wheat genotypes. Out of 12 genotypes, equal number of genotypes (6 each) had higher and lower SG and SRM traits. These genotypes were selected from our previous genome-wide association study for SG and SRM traits. Validation of mapped MTAs have been accomplished by using physiological and gene expression approach. Gene expression analysis of the identified genes in the MTAs region were carried out in these selected contrasting lines in a pot experiment site at Division of Plant Physiology, Indian Agricultural Research Institute (IARI), New Delhi, India. For SG traits, canopy temperature (CT), soil plant analysis development (SPAD) value, leaf senescence rate (LSR) was recorded, whereas for SRM, stem reserve mobilisation efficiency (SRE) was measured. The experiment was carried out in completely randomized design (CRD), under control and combined heat and drought stress (HD) condition. Plants in the control condition (timely sown) were irrigated at their critical phenological stages throughout the cropping period, while under combined stress (50 days late sown), irrigation was withheld at the flowering stage to impose drought stress. Candidate genes found in the overlapping region and within the region of 100 Kb intervals flanking either side of the associated markers were identified through BioMart tool in Ensemble Plants platform. Real-time gene expression analysis was performed on SG-associated genes in the flag leaf and SRM- associated genes in the peduncle. Phenotypic assessment showed that there was significant genotypic variation for the SG and SRM traits and yield. Low SG and SRM performing genotypes showed around 27% and 37% faster leaf senescence rate (LSR) than high SG and SRM performing genotypes under control and HD conditions, respectively, which confirming to our mapped MTAs for SG and SRM traits. HD3366 showed highest stem reserve mobilisation efficiency (SRE) of around 85% under combined stress, while lowest of around 27% was recorded in MP1369 under control condition. Thousand grain weight (TGW) showed negative association with LSR, while positive correlation with SRE. However, highest relative gene expression of *cytokinin dehydrogenase 11-like* (*TaCKX11*) was recorded in low performing SG and SRM genotypes, while lowest expression was recorded in high performing SG and SRM genotypes. Expression analysis of candidate genes like *protein phosphatase 2C* (*TaPP2C*), *TaCKX11*, *protein detoxification 40-like* (*TaPD*), *F-box protein* (*TaFBP*) and *pentatricopeptide repeat* (*TaPPR*) were associated with leaf senescence (SG- linked). Genes linked with SRE, such as *serine/threonine-protein kinase 2* (*TaSK2*) and *wall-associated receptor kinase 4- like* (*TaWAK*) exhibited the highest expression levels during 12 days after anthesis, suggesting their involvement in enhanced carbon reserve mobilization to the grain under stress conditions. Our study confirmed the association of mapped markers and its linked traits, which can be used in further marker-assisted selection (MAS) using efficient breeding tools.

## Introduction

Wheat (*Triticum aestivum L*.) anthesis and grain filling stages are frequently subjected to high temperature and drought stress in the majority of the world’s wheat growing areas, resulting in severe yield losses [1,2]. Wheat growth and productivity is more severely impacted by the combination of terminal drought and heat stress than by either stress alone [3]. In sensitive genotypes, terminal heat, and drought stress may cause early senescence, which shortens the grain filling phase and lowers assimilate synthesis during grain filling [4]. Heat and drought both cause physiological responses in plants, and the overall effect of these two stresses on plants is additive, which leads to increased damage under combined stress conditions [5]. In order to achieve stress tolerance, breeding programs must include morphological and physiological features that can maintain grain-filling ability under abiotic stress environments [6]. Staygreen (SG) as source of current assimilation on one hand and stem reserve mobilization (SRM) on other hand may be mutually exclusive [7]. Flag leaf photosynthesis, chlorophyll content, SG [8,9] and the remobilization of accumulated water-soluble carbohydrates (WSCs) from the internodes of stem to sink [10] are positively correlated with wheat grain yield.SG traits, soil and plant analysis development (SPAD) value has a various benefit, such as quick simple measurement, a non-destructive approach, and the ability to calculate the average value across multiple samples [11]. Canopy temperature (CT), has a direct association with transpiration or evaporation from the leaf surface, and also closely connected with stomatal conductance [12,13]. Chlorophyll concentration in leaf is considered as one of the SG traits, which is linked to stress tolerance [14,15]. Chlorophyll content affects plant dry matter and yield in wheat under stress [16] and also it linked to transpiration efficiency, suggesting that it contributes to the process of stress tolerance [17]. Furthermore, the visual observation of the SG trait has been connected to the maintenance of leaf chlorophyll and photosynthetic capacity [18]. Improved version of the flag leaves senescence scale (FLSS) was used to score senescence [19] and it is used as instant visual indication of staygreen while selecting a field for tolerance of heat stress [15]. Regarding SRM, the main carbon stores in wheat stems are fructans, which are required for grain filling under abiotic stress condition. WSCs make upto 40% of the weight of the stem, of which 85% are fructans. SRM is a crucial process, supports filling of grains under combined heat and drought stress conditions [20]. Non-defoliated plants showed lower SRM than defoliated plants, under combined drought and heat stress condition higher SRM recorded compared to control condition [21,22]. MTAs identified from our previous study [23] encodes a genes like *protein phosphatase 2C*, *cytokinin dehydrogenase 11*, *protein Detoxification 40-like*, *F-box protein* and *pentatricopeptide repeat* are involved in the regulation of leaf senescence (stay green). Putative candidate genes like *serine/threonine-protein kinase 2* and *wall-associated receptor kinase* are involved in enhanced mobilization of carbon reserve to grain (SRM) under stress condition.

Abscisic acid (ABA) regulates drought resilience by coordinating stomatal closure, osmotic adjustment, and stress-protective gene expression. Under drought stress F-box protein (*TaFBP*) inhibits the ABA dependent closure of stomata [24], *AtFBP7* and *TaFBP*, key factors in plant stress response, enhance translational efficiency under high temperature [25] and water deficit conditions [26], respectively. Protein detoxification 40-like isoform X1 interacts with Hsp60, Hsp70, and Hsp90 [27] and plants express these heat shock proteins in response to various stresses like heat, salinity, water stress, oxidative, osmotic and cold stress [28,29]. PSII under stress was more stable when *TaeIF3g* was overexpressed, which was similarly shown with trehalose [30] and *TaeIF5A1* promotes the production of enzymes that scavenge free radicals [31]. Clade protein phosphatase 2C (*PP2C*) serve as ABA signalling co-receptors and also play a crucial role in ABA dependent responses to stress, such as salt, drought, and cold [32–35]. ABA- responsive elements binding factors (ABFs) help to regulate ABA signalling through negative feedback by facilitating the quick activation of group A *PP2C* genes by ABA [36]. E3 ubiquitin-ligases were regulates numerous physiological functions, such as auxin and ABA signalling, adaptive pathway to nitrogen limitation [37,38] and ABA-dependent response to drought [39]. Plasmid encoded polymerase (PEP) driven chloroplast expression of genes is regulated by the Arabidopsis pentatricopeptide repeat (*PPR*) protein DELAYED GREENING1 (DG1) during the early stages of chloroplast development, and the dg1 mutant exhibits a delayed greening phenotype [40]. The primary enzyme that deactivates cytokinin is *cytokinin oxidase/dehydrogenase* (*CKX*), which cleaves the N6 side chains of cytokinin irreversibly to produce adenine or adenosine. Targeting CKX directly or indirectly may have an impact on cytokinin homeostasis [41]. *OsCKX11* coordinates the source-sink relationship in rice by controlling photosynthesis and grain number [42] and *TaCKX11* highly expressed in leaves, it regulates the leaf senescence in wheat [43], therefore influencing SG traits.

ABA is recognized by pyrabactin resistance (PYR)/PYR1-like (PYL)/regulatory factors of ABA receptor (RCAR) receptors, which inhibit PP2C activity, subsequently activating Sucrose non-fermenting-1-related protein kinase 2 (*SnRK2*) protein kinases. These kinases then phosphorylate and activate various proteins that play a role in controlling stomatal closure [44]. *SnRK2* controls the ABA pathway, ABA-mediated gene expression, and stomatal closure [45,46] and provide drought tolerance in rice [47]. Additionally, ABA enhance the stem reserve mobilisation [48] by *SnRK2* depended re-mobilization of WSCs in rice [49]. It was reported that wall-associated receptor kinase 4-like (*WAK*), is a signalling system that controls cell elongation and expansion [50]. In addition to regulating plant growth, development, and adaptability to abiotic stress and hormones, *WAK* is linked to pathogen stress responses [51]. In addition to that *WAK* controls sucrose phosphate synthase expression, altering the sugar-sink relationship [52].

The main objective of the current study is to evaluate the contrasting wheat genotypes under combined HD stress condition and also to analyse the genotypic variability for the SG and SRM traits. To assess the degree of genetic diversity in linear rate of grain growth and the percentage contribution of stem reserves to grain yield for the same wheat genotypes under control and HD conditions. Furthermore, to validate the mapped MTAs using selected lines from our previous study [53] by using physiological and gene expression approaches for their possible transfer to elite wheat genotypes through marker-assisted backcross programme and others efficient breeding techniques.

## Materials and Methods

### Plant materials and growth conditions

Twelve wheat genotypes were used in the present investigation. Out of which 6 genotypes were having high stay-green (SG) and stem reserve mobilization (SRM) traits (DBW309, HD3366, HPW473, HI1654, HI1648, PBW833) and other 6 genotypes were having low SG and SRM traits (AKAW5099, DBW110, GW521, MACS6768, MP1369, MP1372). This wheat genotypes were selected based on our previous experiment during the 2022−2023 rabi season [53]. The multi-trait genotype-ideotype distance index (MGIDI) under various environmental stress conditions was used to select the set of contrasting genotypes [54]. The selected wheat genotypes were evaluated under control and combined heat and drought stress (HD), in pot culture at the ICAR-IARI in New Delhi, India, during the rabi season in 2023–24. The experiment was conducted in factorial completely randomized design (CRD), with six replications of each genotype. For getting terminal combined heat and drought stress, there was an almost 50-day interval between the timely sown (control) and late sown (HD) crops. During the cropping period, plants in the irrigated condition received constant irrigation, whereas under combined stress restricted irrigation was given after the booting stage to impose drought stress at the anthesis stage. We monitored soil moisture using tensiometers and induced drought stress by withholding water until the readings reached −50 kPa (moderate drought stress for wheat). At this point water was applied until the tensiometer reading reached −15 kPa (field capacity for the exact soil type) to maintain uniform drought stress. A control group of plants was maintained with tensiometer readings between −10 and −20 kPa throughout the experiment.

The wheat crop growing season (2023−24), minimum and maximum temperatures (°C) and rainfall (mm) were displayed in S1 Fig. Under both control and HD conditions, the soil moisture content (SMC%) was measured at regular intervals from the anthesis stage to physiological maturity. 25g of fresh soil samples were removed from the rooting zone of potted plants and dried at 105°C in a hot oven. In accordance with Faulkner et al. (1989), the SMC was computed as


$$\text{SMC}\;(\%)=\frac{\text{Soil}\;\text{fresh}\;\text{weight}\;(\text{FW})-\text{Soil}\;\text{dry}\;\text{weight}\;(\text{DW})}{\text{Soil}\;\text{dry}\;\text{weight}\;(\text{DW})}\times \text{100}$$

At the anthesis stage, the average soil moisture content recorded in the root zone was 23.61% for the control condition, and 12.28% for the HD condition.

### Phenotyping of wheat genotypes for SG

Plant sampling and SG parameters were recorded under control and HD conditions. Physiological observations included Soil and plant analysis development (SPAD) value was determined by the method of [55]. Using a portable Minolta SPAD-502 chlorophyll analyzer, SPAD values were measured in the center of flag leaves (Minolta Camera Co. Ltd. Osaka, Japan). A handheld infrared thermometer called a Temperature Gun (Model MT 4, HTC Instruments, Mumbai, India) was used to measure the canopy temperature (CT) by aiming it at the canopy leaves at a 45° angle to the horizontal. Phenotyping for leaf senescence was carried out through visual scoring based on a range from 0 to 10, dividing the percentage of predicted area that was dead by time duration in days as per the method [19,23,56].


$$\text{LSR}=\frac{\text{Initial}\;\text{score}\;-\;\text{Final}\;\text{score}}{\text{No}.\;\text{of}\;\text{scoring}\;\text{days}}$$

For SPAD value, CT, total chlorophyll and carotenoid estimation observations were recorded at stage-1: 50% anthesis, stage-2: 10 days after anthesis (DAA), and stage-3: 20 DAA under control condition. In case of HD condition observations were recorded stage-1: at 50% anthesis, stage-2: 5 DAA, and stage-3: 10 DAA. Yield related observation were recorded at the time of harvest, such as thousand grain weight (TGW), was also recorded to classify contrasting wheat genotypes.

### Total chlorophyll and carotenoid estimation

The dimethyl sulfoxide (DMSO) method was used to measure the total amount of carotenoid and chlorophyll [57]. To the test tubes with 7 ml of DMSO, 25 mg of fresh leaf samples (small discs) were added. Tubes were stored at 65°C for four hours in the dark. The absorbance of the given volume of fluid containing a known amount of leaf tissue was measured at two different wavelengths (663 and 645 nm) to determine the amount of chlorophyll and at 480 nm for total carotenoids. Arnon’s formula was used to calculate chlorophyll content [58].

### Phenotyping of wheat genotypes for SRM

The water-soluble carbohydrates (WSCs) in the stem were measured at 12 DAA and physiological maturity (PM). 50 mg of sample was boiled at 80 °C on a boiling water bath with 5 ml of 80% ethanol, and then performed two extractions at 70 °C with 5 ml distilled water for at least 1 hr. The extract was separated from the supernatant by centrifuging it for 10 minutes at 4500 rpm at room temperature. Anthrone reagent was prepared by dissolving 2 mg of anthrone in 100 ml of ice-cold concentrated H_2_SO_4_ [59] and then we, added 4 ml solution of anthrone reagent to 1 ml of sample. At 630 nm, the absorbance was measured. The calculation was done by using the standard curve equation (y = 0.0066x + 0.0372, R² = 0.9896). We prepared a series of known glucose concentrations (standards), added the anthrone reagent, and then measure the absorbance at 630 nm. By using spectrophotometer readings, we calculated glucose standard curve. Stem reserve mobilization efficiency (SRE), was calculated by utilizing the following formulae [10]:


$$\text{SRE}=\frac{\text{Stem}\;\text{WSCs}\;\;\text{at}\;\text{12}\;\text{DAA}\;-\;\text{Stem}\;\text{WSCs}\;\;\text{at}\;\text{PM}}{\text{Stem}\;\text{WSCs}\;\text{at}\;\text{12}\;\text{DAA}\;}\times \text{100}$$

### *In silico* expression analysis

The putative candidate genes reported in our previous investigation were used for in-silico and in-vivo gene expression analysis [53]. The potential candidate genes found in the overlapped region within the 100 Kb intervals associated with both sides of the linked marker were identified using the BioMart tool in the ensemble plants platform. Furthermore, Wheat Expression database [60] was used to find their expression patterns in different plant parts. According to earlier publications, the function of the potential candidate genes that were discovered in the regulation of SG and SRM traits were also analysed.

### Reverse transcriptase PCR analysis

To study the expression patterns of SG-linked genes, flag leaf samples were collected for RNA isolation in three cropping stages, i.e., at 50% anthesis, 10 DAA, 20 DAA under control condition, while under combined stress, sampling was done at 50% anthesis, 5 DAA, 10 DAA for gene expression analysis. However, peduncles of the main tillers were collected at 12 DAA under control and combined stress to study the SRM-linked genes. We used Sigma’s Spectrum Plant Total RNA Kit for RNA isolation. DNAse I (Thermo Scientific, USA) was used to digest DNA present in the sample before reverse transcription. Nanodrop readings were taken for quantification of nucleic acids, where 2 µl of RNA was used for absorbance readings at A260/A280. Thermo Scientific Verso cDNA synthesis kit was utilized for the amplification of complementary DNA (cDNA), from RNA samples. Consequently, primers were designed using PrimerQuestTM tools available at Integrated DNA Technology (IDT) software [61], which were given in S1 Table. A Step One Plus TM real-time detection system was used to conduct a reverse transcriptase PCR experiment in order to measure gene expression under both control and HD conditions. The comparative Ct technique was used to calculate fold changes in gene expression (2-ΔCt) [62], relative to treated sample, where ΔCt = [Ct target gene- Ct reference gene]. Actin was set as a reference gene (housekeeping gene) for normalization of gene expression data.

## Results

### Phenotyping for staygreen (SG)

**Fig 1:**
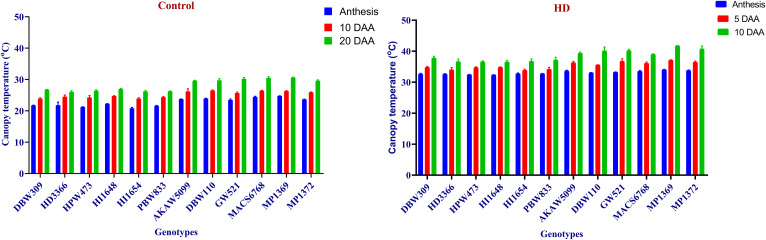
Canopy temperature (CT) value under Control and combined stress (HD) condition. Under control conditions, the colors blue, red, and green indicate CT values at anthesis, 10 days after anthesis (DAA), and 20 DAA, respectively. In contrast, under the HD condition, these colors correspond to CT values at anthesis, 5 DAA, and 10 DAA, respectively.

**Fig 2:**
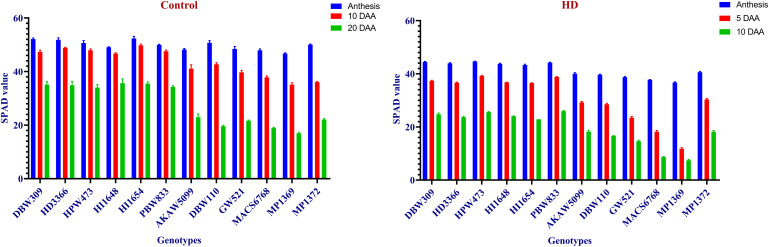
Soil Plant Analysis Development (SPAD) value under Control and HD condition. Blue, red and green colors representing the SPAD values at Anthesis, 10 DAA (Days after anthesis) and 20 DAA, respectively under control condition. Under HD condition these colors representing the SPAD value at Anthesis, 5 DAA and 10 DAA, respectively.

**Fig 3:**
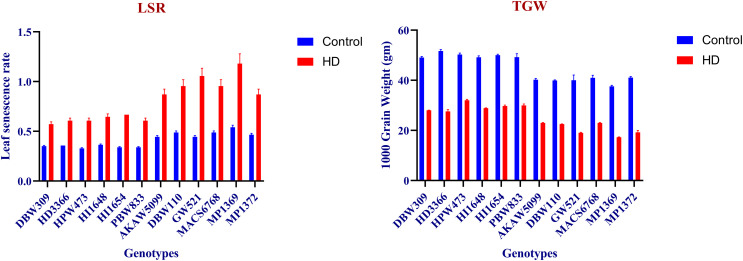
Leaf senescence rate (LSR) and Thousand grain weight (TGW) under Control and combined stress (HD) condition. Blue and red color representing the control and HD condition, respectively.

From the study, CT was recorded lowest 20.8 under control, while highest of 41.75 was observed under HD condition. Under control condition maximum CT recorded in MP1369 at 20 DAA, whereas minimum observed in HI1654 at anthesis. In case of HD condition, HI1648 showed lowest CT at anthesis, while highest was recorded in MP1369 at 10 DAA (Fig 1). Similarly, SPAD value of 12 wheat genotypes were showed significant difference between 6 high SG and SRM traits genotypes and low SG and SRM performing genotypes. Highest SPAD value observed in HI1654 at anthesis, while lowest was recorded in MP1369 at 20 DAA under control condition. In case of HD condition, HPW473 showed highest SPAD value at anthesis, whereas lowest was recorded in MP1369 at 10 DAA (Fig 2). LSR showed highest in MP1369 (1.18) under HD, whereas minimum recorded in HPW473 (0.327) under control condition. Low SG and SRM performing genotypes showed around 27% and 37% faster LSR than high SG and SRM performing genotypes under control and HD conditions respectively (Fig 3). TGW recorded highest of 51.65 gm in HD3366 under control, while lowest of 17.3 gm observed in MP1369 under HD condition. High SG and SRM performing genotypes showed around 20% and 18% more TGW than low SG and SRM performing genotypes under control and HD conditions, respectively (Fig 3).

### Chlorophyll a and b, chl a/b, total chlorophyll, total carotenoids

**Fig 4:**
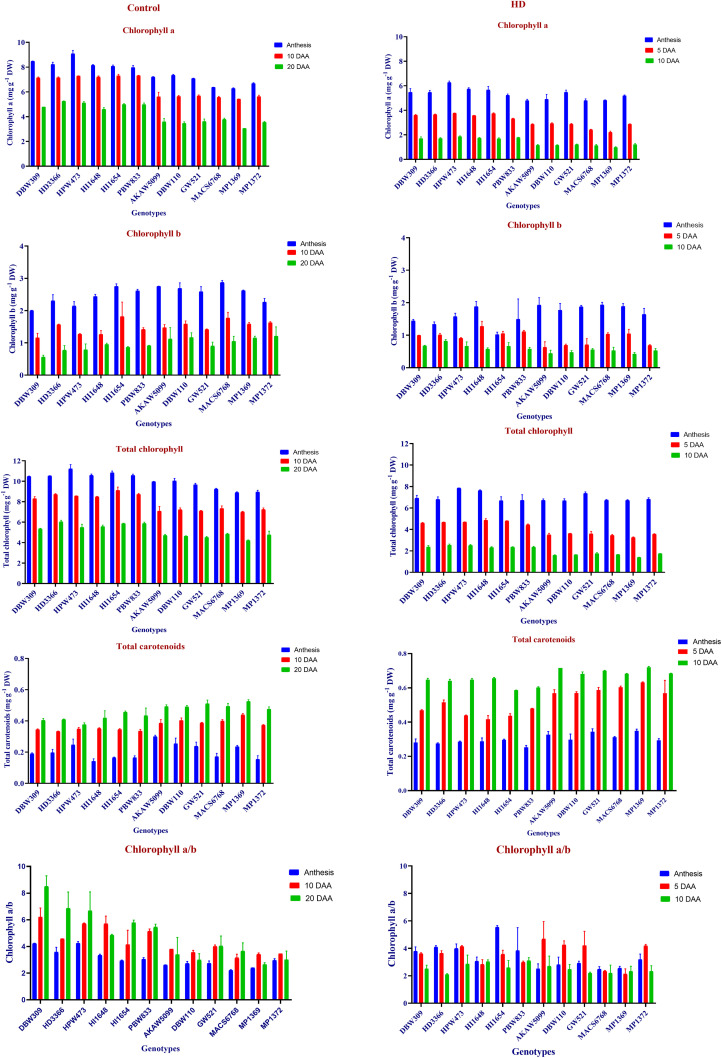
Chlorophyll a, b, chl a/b, total chlorophyll and total carotenoids content under Control and HD condition. Under control conditions, the colors blue, red, and green indicate at anthesis, 10 days after anthesis (DAA), and 20 DAA, respectively. In contrast, under the HD condition, these colors representing at anthesis, 5 DAA, and 10 DAA, respectively.

Maximum chlorophyll a content was recorded under control condition in HPW473 at anthesis (9.08 mg g^-1^ DW), whereas minimum was observed in MP1369 at 20 DAA (3.05 mg g^-1^ DW). In case of HD condition, MP1369 showed lowest chlorophyll a content of 0.97 mg g^-1^ DW at 10 DAA, while highest content of 6.27 mg g^-1^ DW was showed in HPW473 at anthesis (Fig 4). MACS6768 showed highest chlorophyll b content of 2.87 mg g^-1^ DW at anthesis stage under control condition, while the least was recorded in HPW473 at 20 DAA (0.37 mg g^-1^ DW). Under HD condition, AKAW5099 and MACS6768 showed maximum chlorophyll b content of 1.92 and 1.93 mg g^-1^ DW respectively at anthesis, while minimum was recorded in MP1369 at 10 DAA as 0.42 mg g^-1^ DW (Fig 4). Under control condition, HPW473 recorded highest total chlorophyll content of 11.22 mg g^-1^ DW at anthesis stage, while the lowest was observed under MP1369 at 20 DAA (4.2 mg g^-1^ DW). HPW473 showed the highest total chlorophyll content of 7.85 mg g^-1^ DW at anthesis stage under HD condition, while the least content of 1.56 mg g^-1^ DW was recorded in MP1369 at 10 DAA (Fig 4). HI1648 showed minimum total carotenoids at anthesis stage (0.14 mg g^-1^ DW), while maximum was observed in MP1369 at 20 DAA (0.52 mg g^-1^ DW) under control condition. Under HD, in MP1369 recorded highest total carotenoids at 10 DAA (0.72 mg g^-1^ DW), whereas lowest was showed in PBW833 at anthesis stage was 0.25 mg g^-1^ DW (Fig 4). Under control condition, DBW309 recorded highest Chl a/b of 9.06 at 20 DAA, while the lowest was observed under MACS6768 at anthesis stage (2.18). AKAW5099 showed the highest Chl a/b ratio of 5.57 at 5DAA under HD condition, while the least ratio of 1.87 was recorded in MP1369 at 5 DAA (Fig 4).

### Phenotyping for stem reserve mobilization (SRM)

**Fig 5:**
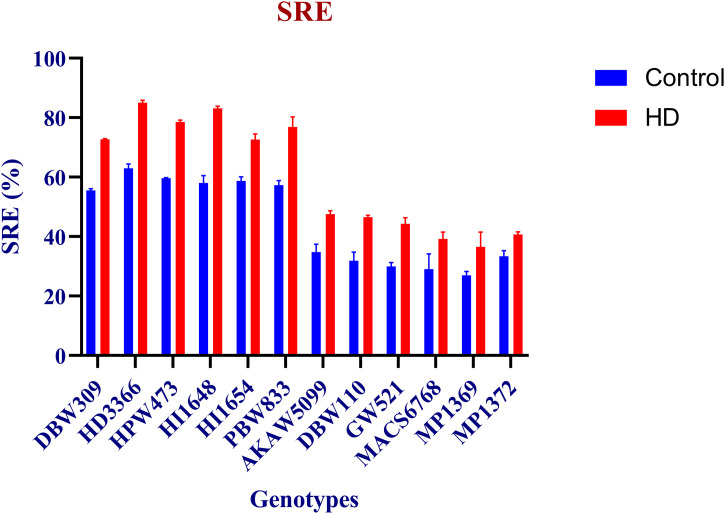
Stem reserve mobilization efficiency (SRE) percentage under Control and HD condition. Blue and red color indicates the control and HD condition, respectively.

SRE was measured through stem water-soluble carbohydrates content in 12 wheat genotypes at 12 DAA and physiological maturity (PM) under control and HD conditions. Under control, highest WSCs content was recorded in HI1654 (30.17 mg g^-1^ DW), while lowest of 14.73 mg g^-1^ DW observed in MACS6768 under HD condition at 12 DAA. WSCs at PM stage recorded minimum of 3.27 mg g^-1^ DW in HD3366 under combined stress, whereas maximum observed in MP1369 (18.99 mg g^-1^ DW) under control condition (S2 Fig). SRE showed highest mean value in all high performing SG and SRM genotypes compared to low SG and SRM performing genotypes under both control and HD condition. HD3366 showed highest SRE of around 85% under HD, while lowest of around 27% was recorded in MP1369 under control condition. High-performing genotypes showed around 27% and 36% more SRE than low-performing genotypes under control and HD conditions respectively (Fig 5).

### *In-silico* expression analysis of putative candidate genes

*In-silico* expression patterns of identified putative candidate genes for canopy temperature (TraesCS3B02G563100, TraesCS3D02G506600, TraesCS5A02G036300, TraesCS2D02G214200 and TraesCS7A02G017800) on different plant parts were investigated (S3A Fig). For SPAD value, in-silico gene expression of identified genes (TraesCS3D02G018100, TraesCS3D02G018200 and TraesCS2A02G378300) were showed in S3B Fig and the expression pattern of NDVI genes (TraesCS5D02G385000 and TraesCS5D02G385125) also investigated (S3C Fig). The expression pattern of genes identified for SRM (TraesCS6B02G458200, TraesCS2A02G056100, TraesCS2A02G056800, TraesCS2D02G052000 and TraesCS2D02G053300), on shoot, leaves and spike were founded through Wheat Expression database (S3D Fig). The roles of the putative candidate genes that were identified in the control of SG and SRM traits in accordance with previous publications were listed in Table 1.

**Table 1:** The role of the identified putative candidate genes in the regulation of staygreen and stem reserve mobilization traits.

Traits	MTAs reported [23]	Transcript stable ID	Position	Gene name	Function	Reference
**CT**	AX-94724702	TraesCS3B02G563100.2	3B: 796,008,219–796,019,084	Protein DETOXIFICATION 40-like isoform X1 (*TaPD*)	Protein folding and prevent non-native proteins aggregation during abiotic stress conditions	[27]
	AX-94613383	TraesCS3D02G506600.2	3D: 593,345,514–593,348,574	Eukaryotic translation initiation factor 3 subunit M-like (*TaEIF3*)	Increase the photosynthetic efficiency and protect plants from photooxidative stress under drought	[63]
	AX-94556600	TraesCS5A02G036200.1	5A: 33,126,001–33,130,778	Chloroplast stem-loop binding protein (*TaCSL*)	Controls the expression of chloroplast genes	[64]
	AX-95192394	TraesCS2D02G214200.1	2D: 175,591,674−175,595,822	Probable protein phosphatase 2C (*TaPP2C*)	ABA dependent stress responses	[32]
	AX-94780124	TraesCS7A02G017800.1	7A: 7,628,704−7,630,503	F-box protein (*TaFBP*)	Regulates leaf senescence by degradation of target substrates	[65]
**SPAD**	AX-94636264	TraesCS3D02G018100.1	3D: 6,140,574−6,142,644	Putative E3 ubiquitin-protein ligase SINA-like 9 (*TaE3-ubiquitin*)	Post-translational protein modification	[66]
		TraesCS3D02G018200.1	3D: 6,146,779−6,149,852	Putative pentatricopeptide repeat-containing protein (*TaPPR*)	Chloroplast development in early growth stage	[40]
		TraesCS2A02G378300	2A:621182540–621186230	*Cytokinin dehydrogenase 11-like* (*TaCKX11*)	Catalyses irreversible cytokinin degradation	[41]
**NDVI**	AX-94516324	TraesCS5D02G385000.1	5D: 454,169,953−454,173,859	Single myb histone 5-like (*TaMYB*)	ABA dependent stomatal closure	[67]
		TraesCS5D02G385125.1	5D: 454,202,647−454,205,436	Cationic amino acid transporter 1-like (*TaCAAT-like*)	Enhance the proline content to mitigate drought stress	[68]
**SRE**	AX-95126447	TraesCS2A02G056100.1	2A: 23,958,801−23,961,127	UDP-glucosyltransferase (*TaUGT*)	Regulation of grain size	[69]
	AX-94844376	TraesCS2D02G051800.2	2D: 19,603,312−19,624,800	ABC transporter G family member 43-like (*TaABC*)	Positive effect on wheat grain formation	[70]
	AX-95151743	TraesCS2D02G053300.1	2D: 20,768,191−20,770,988	Serine/threonine-protein kinase 2 (*TaSK2*)	ABA dependent fructan hydrolysis	[71]
	AX-94740659	TraesCS6B02G458200.1	6B: 713,458,733−713,462,914	Wall-associated receptor kinase 4-like (*TaWAK*)	Regulates the expression of sucrose phosphate synthase	[52]

### Relative gene expression of selected genes for CT

**Fig 6:**
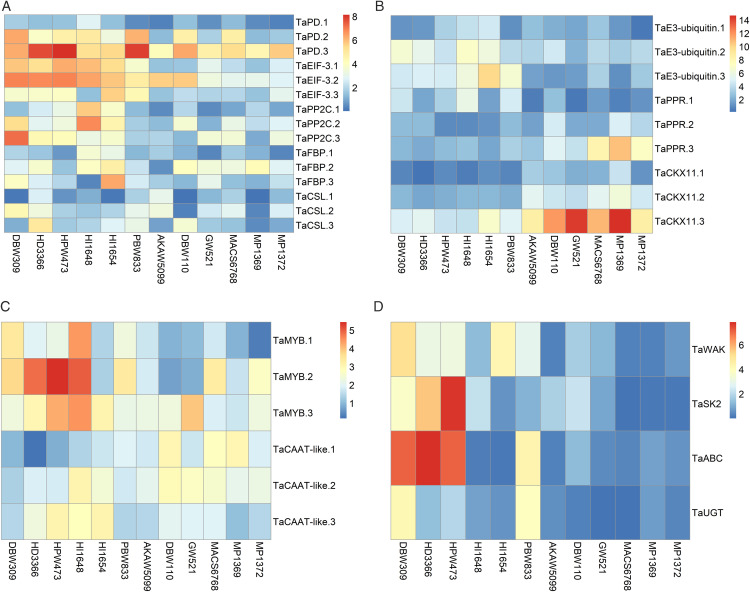
A. Heatmap of relative gene expression for canopy temperature. *TaPD* (TraesCS3B02G563100), *TaEIF-3* (TraesCS3D02G506600), *TaPP2C* (TraesCS2D02G214200), *TaFBP* (TraesCS7A02G017800), *TaCSL* (TraesCS5A02G036300); after gene name numbers 1, 2, and 3 represents the expression levels during stage-1 (50% Anthesis), Stage-2 (5DAA), Stage-3 (10 DAA), respectively. B. Heatmap of relative gene expression for Soil and plant analysis development (SPAD) value. *TaE3 ubiquitin* (TraesCS3D02G018100), *TaPPR* (TraesCS3D02G018200), *TaCKX11* (TraesCS2A02G378300); after gene name numbers 1, 2, and 3 represents the expression levels during stage-1 (Anthesis), stage-2 (5DAA), stage-3 (10 DAA), respectively. C. Heatmap of relative gene expression for Normalized difference vegetation index (NDVI). *TaMYB* (TraesCS5D02G385000), *TaCAAT-like* (TraesCS5D02G385125); after gene name numbers 1, 2, and 3 represents the expression levels during stage-1 (Anthesis), stage-2 (5DAA), stage-3 (10 DAA), respectively. D. Heatmap of relative gene expression for Stem Reserve Mobilisation Efficiency (SRE). *TaWAR* (TraesCS6B02G458200), *TaSK2* (TraesCS2D02G053300), *TaABC* (TraesCS2A02G056800), *TaUGT* (TraesCS2A02G056100) expression levels in peduncle.

Relative gene expression of Protein detoxification 40-like (*TaPD*) in 6 contrasting wheat genotypes showed a gradual increase from anthesis to 10 DAA under HD condition compared to control. HD3366 (7.678 folds), PBW833(7.904 folds) and HPW473 (8.152 folds), showed high expression at 10 DAA under HD as compared to control condition. Lowest expression was observed in AKAW5099 and MP1369 by 4.174 folds and 4.446 folds respectively. Eukaryotic translation initiation factor 3 subunit M-like (*TaEIF3*), showed enhanced expression from anthesis to 5DAA, but at 10 DAA there was a reduction in expression. Highest expression of 6.916 folds was recorded in HPW473 at 5DAA under HD compared to control. Under HD condition protein phosphatase 2C 64 (*TaPP2C*) gene expression increased from anthesis to 5DAA, but reduced expression was recorded during later stages. DBW309 (7.242 folds) showed an enhance expression from anthesis to 10 DAA. The F-box protein (*TaFBP*), showed a similar pattern, but DBW309 (3.936 folds) and HI1654 (6.358 folds) showed enhance expression at 10 DAA, while least expression of 1.203 folds was recorded in MP1369 under HD compared to control. Highest Chloroplast stem loop binding protein (*TaCSL*) gene expression was recorded in HD3366 (5.063 folds) at 10 DAA, while lowest of 0.033 folds observed in MP1369 at anthesis stage (Fig 6A). Relative gene expression of all the studied genes Mean and standard deviation was given in S2 Table.

### Relative gene expression of selected genes for SPAD value

Expression pattern of E3 ubiquitin-protein ligase SINA-like 9 (*TaE3-ubiquitin*) showed enhance pattern from anthesis to 5DAA, but at 10 DAA observed reduce expression in some genotypes. However highest expression was recorded in HI1654 at 10 DAA (10.026 folds) under HD compared to control, while lowest of 0.378 folds showed in MP1372 at anthesis stage. Pentatricopeptide repeat-containing protein (*TaPPR*) showed enhance expression in high SG and SRM performing genotypes at anthesis, whereas enhanced expression was observed in low SG and SRM performing genotypes at 10 DAA under HD condition compared to control condition. MP1369 and MACS6768 recorded highest *TaPPR* gene expression of 10.683 folds and 8.295 folds respectively. Relative gene expression of *cytokinin dehydrogenase 11-like* (*TaCKX11*) in 6 contrasting wheat genotypes showed a gradual increase from anthesis to 10 DAA under HD compared to control condition (S2 Table). MP1369 and DBW110 genotypes showed the highest *TaCKX11* gene expression of 14.702 folds and 11.919 respectively at 10 DAA, while the lowest of 0.175 folds recorded in HD3366 at anthesis (Fig 6B).

### Relative gene expression of selected genes for NDVI

Single MYB histone 5-like (*TaMYB*) showed enhance expression from anthesis to 5DAA, but at 10 DAA we observed reduce expression in high SG and SRM traits genotypes, whereas increase in expression was observed in low SG and SRM performing genotypes at 10 DAA under HD compared to control condition. Relative gene expression of cationic amino acid transporter 1-like (*TaCAAT-like*) showed a gradual increase from anthesis to 10 DAA in high SG and SRM performing genotypes. Furthermore, MP1369 genotypes showed 1.079-fold decreased expression, but HI1654 showed approximately 3.08-fold increase in expression under HD in comparison to control at 10 DAA (Fig 6C).

### Relative gene expression of selected genes for SRE

Relative gene expression of wall-associated receptor kinase 4-like (*TaWAK*) was found to be 5.13 folds and 4.26 folds higher under HD compared to control in DBW309 and HI1654 respectively. However, in AKAW5099 and MP1369 showed a reduce gene expression by 0.266 folds and 0.299 folds respectively. Serine/threonine-protein kinase (*TaSK2*) showed high gene expression of 7.77 folds and 5.6 folds in HPW473 and HD3366 respectively under HD compared to control condition. However, the relative expression was found to be reduced by 0.08 folds in MACS6768 under HD condition. ABC transporter C family member 4-like (*TaABC*) showed enhance expression in DBW309 (7.092 folds), HD3366 (7.860 folds) HPW473 (7.126 folds), and DBW309 (7.092 folds), compared to low SG and SRM performing genotypes under HD condition (S2 Table). UDP-glucosyltransferase (*TaUGT*) showed high gene expression of 4.19 and 3.88 folds in DBW309 and PBW833 respectively, under HD compared to control condition. Whereas, reduced expression was observed in to low SG and SRM performing genotypes (Fig 6D).

## Discussion

### Phenotypic evaluation of SG and SRM traits

We observed lesser SPAD value under HD as compared to control condition [72]. Moreover, MP1369 had the lowest SPAD value, indicated its less resilience towards hotter climate. CT was recorded high in low SG and SRM performing genotypes under HD, while low CT was observed in high SG and SRM performing genotypes. Previous studies found that tolerant genotype will have cooler canopies, more open stomata, and deeper roots to reach soil water [73,74]. In addition, previous studies revealed a positive correlation of cooler canopy and higher root dry weight in wheat genotypes [75,76]. CT has been found to be a drought-adaptive feature and has a positive correlation with grain yield [14]. Under both control and HD conditions, enhanced LSR was recorded in low SG and SRM performing genotypes (MP1369) compared to high SG and SRM performing genotypes (HPW473). We observed negative association of LSR and TGW. Similarly, reduced grain yield was observed in non-functional stay-green wheat genotypes compared to functional stay-green wheat genotypes [22,56]. HD3366 showed highest SRE of around 85% under combined stress, while lowest of around 27% was recorded in MP1369 under control condition (Fig 5). Similarly in defoliated plants, stem reserves contributed 24–84% of grain weight [22,77], while under stress, remobilized stem WSCs can contribute 30–50% of grain weight [78].

### Light harvesting pigments

The ability of leaves to photosynthesize depends on its pigments. Chlorophyll absorbs the light energy and transfer, it to the photosynthetic apparatus. In our current study, total chlorophyll decreased gradually after anthesis stage under HD in all the studied genotypes [79]. Studies on potatoes and maize have demonstrated that combination stress reduces chlorophyll content more than independent heat and drought stress [80,81] and similar results were reported in wheat [82]. In addition, reactive oxygen species-induced chloroplast damage is the main source of the decrease in chlorophyll content during stress [83]. We observed enhanced total carotenoid content under combined stress, compared to control condition. It might be due to antheraxanthin and zeaxanthin (carotenoid precursors) content enhanced under drought to prevent photoinhibition in wheat [84].

### Expression analysis of CT

Protein detoxification 40-like (*TaPD*) showed enhance expression in high SG and SRM genotypes (HD3366, PBW833 and HPW473). It involved in maintaining the protein functional shape and inhibit aggregation of non-native proteins [27]. *TaEIF3* showed enhanced expression from anthesis to 5DAA, under drought conditions involved in the protection against photooxidative stress [63] and free radical scavenging [31]. Downregulation of *TaEIF3* genes at late maturity stage might be due to conserve resources as a mechanism of the stress response. Enhanced expression of *TaPP2C* in high SG and SRM performing genotypes showed that it functioned as co-receptor of ABA signalling and ABA-dependent stress responses [32] to mitigate drought stress. *TaFBP* showed enhanced expression at initial stages of grain filling (Anthesis to 5DAA), while reduced expression was observed at late grain filling stages. This might be to conserve resources as part of the stress response mechanism. Moreover, *TaFBP* regulates leaf senescence [65], enhances oxidative [26] and heat stress tolerance [66]. *TaCSL* increased expression was observed under high SG and SRM performing genotypes, regulates transcription and translation of chloroplast-encoded RNAs [85]. Additionally, CSP41B was significantly down regulated under drought stress in Arabidopsis [86].

### Expression analysis of SPAD value

Expression of E3 ubiquitin-protein ligase(*TaE3-ubiquitin*) showed a gradual increase in all 6 contrasting genotypes during grain filling stage. *TaE3-ubiquitin* is involved in eukaryotic post-translational protein modification and also regulates numerous cellular functions [87]. Furthermore, *TaE3-ubiquitin* showed a drought tolerance in Arabidopsis through increased production of ABA [88]. Pentatricopeptide repeat-containing protein (*TaPPR*) showed enhance expression at flowering stage and reduced expression pattern was observed during latter phase. DELAYED GREENING1 (DG1) encodes eight pentratricopeptide repeat domains (chloroplast protein) and it regulates PEP-dependent expression of chloroplast genes in the early stages of chloroplast formation, while mutant showed a delayed greening phenotype [40]. *TaCKX11* (Cytokinin dehydrogenase 11) showed a gradual increase in all 6 contrasting wheat genotypes from flowering to maturity. *TaCKX11* catalyses irreversible cytokinin degradation, which leads to enhanced leaf senescence [41]. Moreover, leaf senescence is an internally controlled degradation process that coincides with the reproductive and grain filling stages [4] by regulating source and sink relation [42]. *TaCKX11* expression was highest in MP1369 and DBW110, which indicate non-SG genotypes, and lowest in HD3366 and HPW473, which indicate SG genotypes.

### Expression analysis of NDVI

Myeloid-associated factor B (*TaMYB*) showed enhanced expression from flowering to initial stage of grain filling, while reduce expression was observed during maturity stage. *MYB* proteins can interact with ABA signaling components, such as protein phosphatases and kinases, to modulate ABA sensitivity and response [89]. Furthermore, *MYB* protein regulate the closure of stomata to improve drought tolerance in Arabidopsis [67,90]. Cationic amino acid transporter 1-like (*TaCAAT-like*) showed enhance expression during grain filling stage in high SG and SRM performing genotypes, while reduced expression was observed in low SG and SRM performing genotypes. Moreover, enhanced expression of *TaCAAT-like* in senescing leaves, and stems showed that, it may be involved in remobilization of stem reserve to grain. Furthermore, *TaCAAT-like* plays crucial role in determining grain production and protein content [68].

### Expression analysis of SRE

During the wheat grain filling stage, the crop experiences simultaneous heat and drought stress. Stem reserves play a crucial role in providing WSCs for grain filling during heat and drought stress conditions that restrict photosynthesis. Upregulation of PPR5 and endoglucanse-8 like genes regulating stem reserve mobilization was observed in our previous study [91]. *TaWAK* was highly expressed under stress condition at 12 DAA in peduncle of all high SG and SRM performing genotypes. However, increased stem reserves remobilization to grain was observed in HD3366 and HI1648 under HD. Furthermore, *TaWAK* enhances elongation of cell [50], and also controls the expression of invertase and sucrose-phosphate synthase activity which changes sugar-sinks relation [52]. *TaSK2* showed increase in expression in high performing genotypes compared to low performing genotypes, which may be due to ABA-dependent upregulation of serine/threonine protein kinase genes. Furthermore, previous studies reported that ABA enhances the mobilization of stem reserves [48] by *SnRK2* depended mobilization in rice [49]. UDP-glucosyltransferase (*TaUGT*) recorded enhance expression under HD condition compared to control. *TaUGT* enhances free ABA levels, leading to closing of stomata and enhanced ROS scavenging and detoxification [92]. Moreover, *TaUGT* regulates flavonoid-mediated auxin levels to maintain size of grain and abiotic stress resistant in rice [69]. Expression of *TaABC* enhanced in high SG and SRM genotypes, which might be to maintain activity of sucrose or invertase on cell membranes [70]. Furthermore, transmembrane proteins of the ATP-binding cassette (ABC) family control vacuolar pumps of glutathione conjugates, although they have demonstrated a wide range of roles, such as ion-channel regulation, detoxification, and transport of chlorophyll catabolites [93].

## Conclusion

To fulfil the growing need of food in this period of global climate change, new improved crop varieties must be developed. To accomplish this, identification and its validation of genomic regions or markers linked to contributing physiological traits for grain filling is needed for successful development of improved genotypes using efficient breeding strategies. In this juncture, we validated our mapped MTAs for SG and SRM under combined heat and drought stress condition in wheat. Additionally, significant markers that have been found will assist wheat breeders in accumulating desired allelic combinations for upcoming breeding initiatives. Furthermore, identified superior wheat genotypes can be used as donor parents for SG and SRM traits to elite wheat genotypes for further augmentation in yield. Therefore, knowing the genetic underpinnings of SG and SRM will increase yield in wheat and other crops under this challenging global climate change scenarios.

## Supporting information

S1 Fig Weather parameters depicting maximum temperature (brown lines), minimum temperature (green line), and rainfall (red line) during 2023−24. (JPEG)

S2 Fig Stem water soluble carbohydrates (WSC) content under Control and HD condition at 12 days after anthesis (DAA) and physiological maturity (PM). (TIF)

S3 Fig A *In Silico* gene expression analysis for canopy temperature. B. *In Silico* gene expression analysis for soil plant analysis development value (SPAD). C. *In Silico* gene expression analysis for normalized difference vegetation index (NDVI). D. *In Silico* gene expression analysis for stem reserve mobilization (SRM).
(ZIP)

S1 Table List of forward and reverse primers used for expression analysis of CT, SPAD, NDVI and SRM associated genes. (DOCX)

S2 Table Genotype wise relative fold expression of all the studied genes Mean and SD (standard deviation) for different traits. (XLSX)
