# Supplementary figures and images for: Dissecting the differential performance of contrasting stay-green and stem reserve mobilization wheat (Triticum aestivum L.) genotypes – Validation of GWAS analysis
Source: PLoS One. 2026 Jan 5;21(1):e0339374. doi: 10.1371/journal.pone.0339374 (PMC12768281; doi:10.1371/journal.pone.0339374)

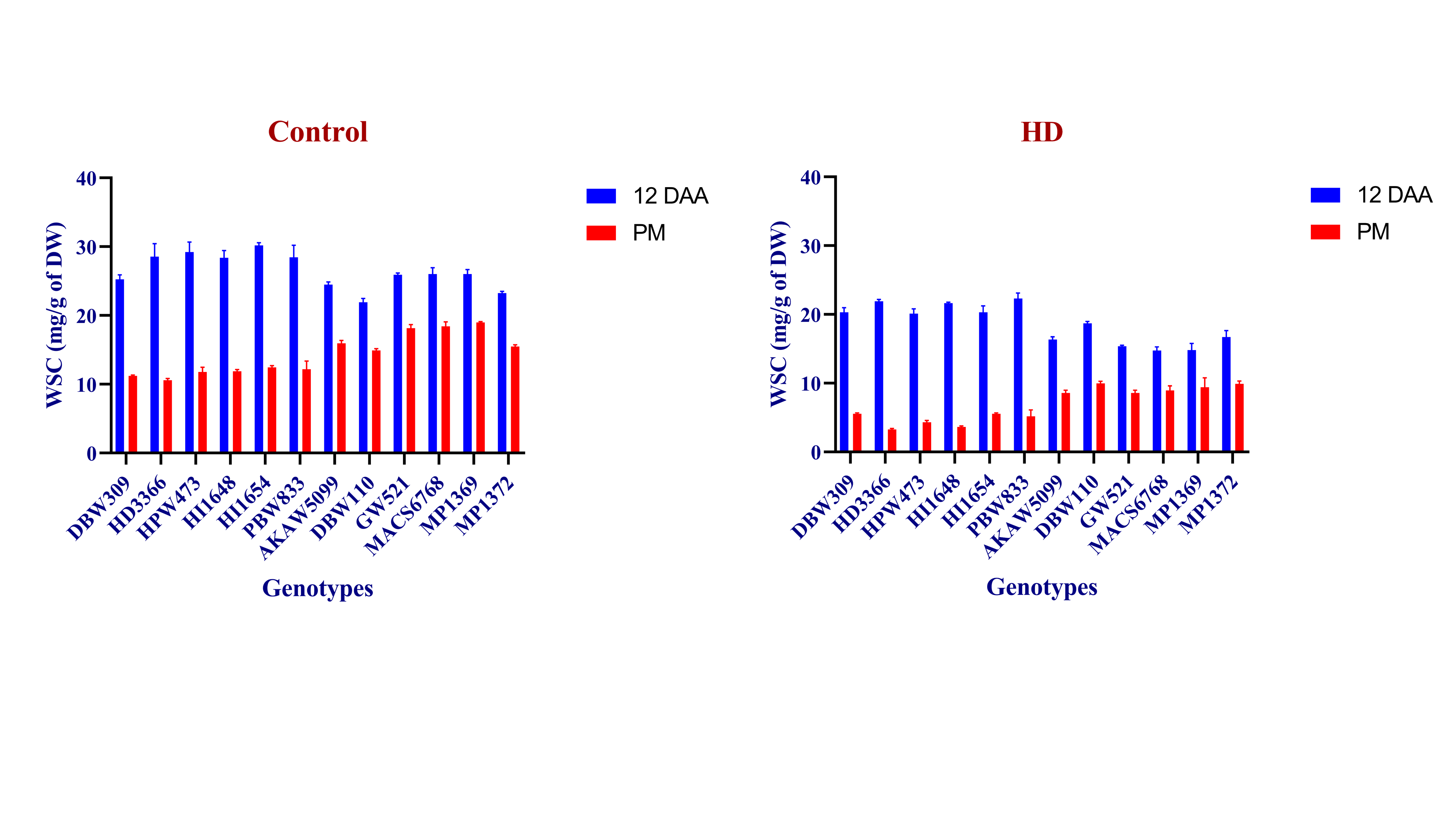

Supplement: S2 Fig — (TIF) [file pone.0339374.s002.tif]

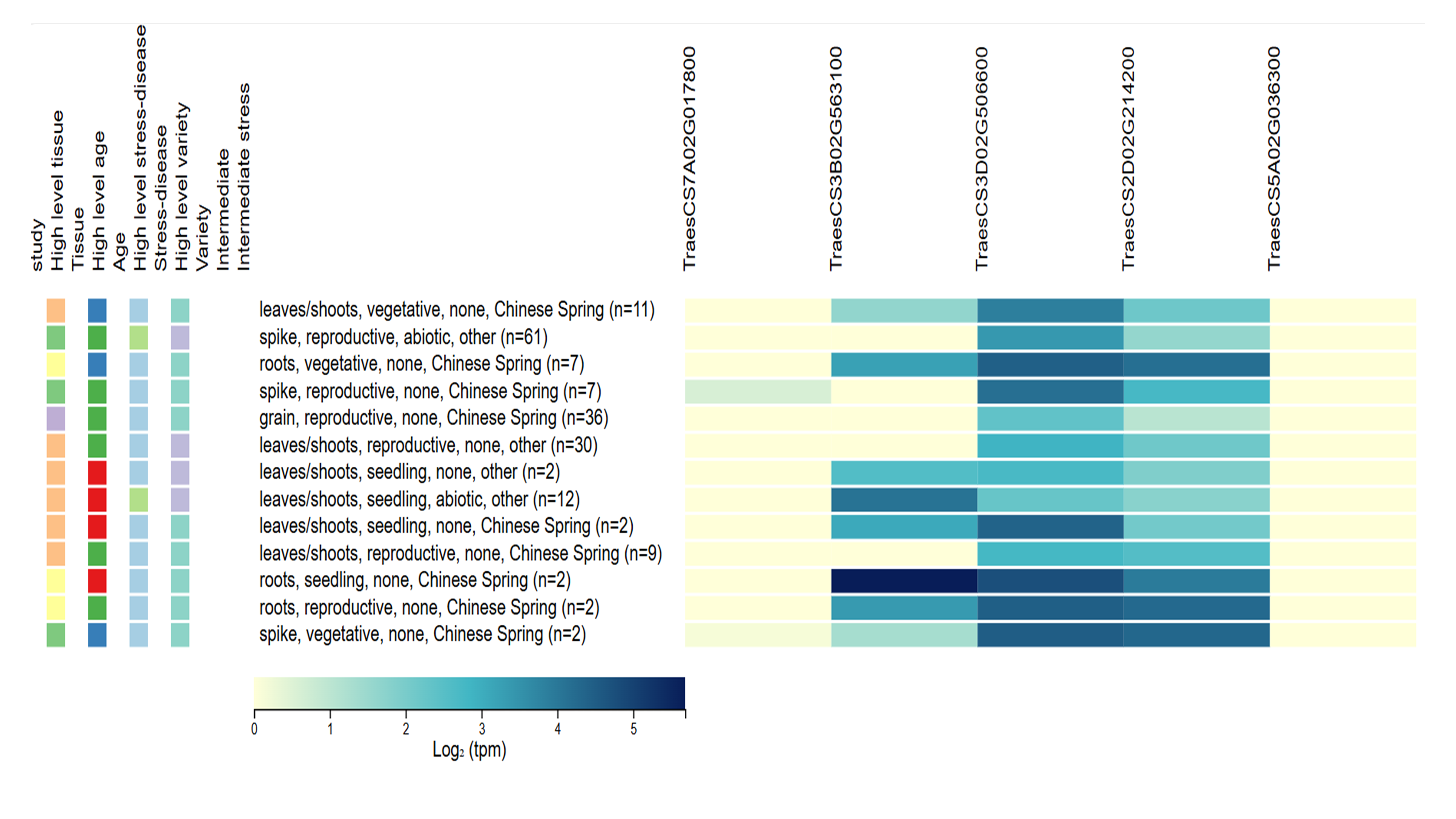

Supplement: S3 Fig — B. In Silico gene expression analysis for soil plant analysis development value (SPAD). C. In Silico gene expression analysis for normalized difference vegetation index (NDVI). D. In Silico gene expression analysis for stem reserve mobilization (SRM). (ZIP) [file pone.0339374.s003.zip › S3A_ Fig.tif]

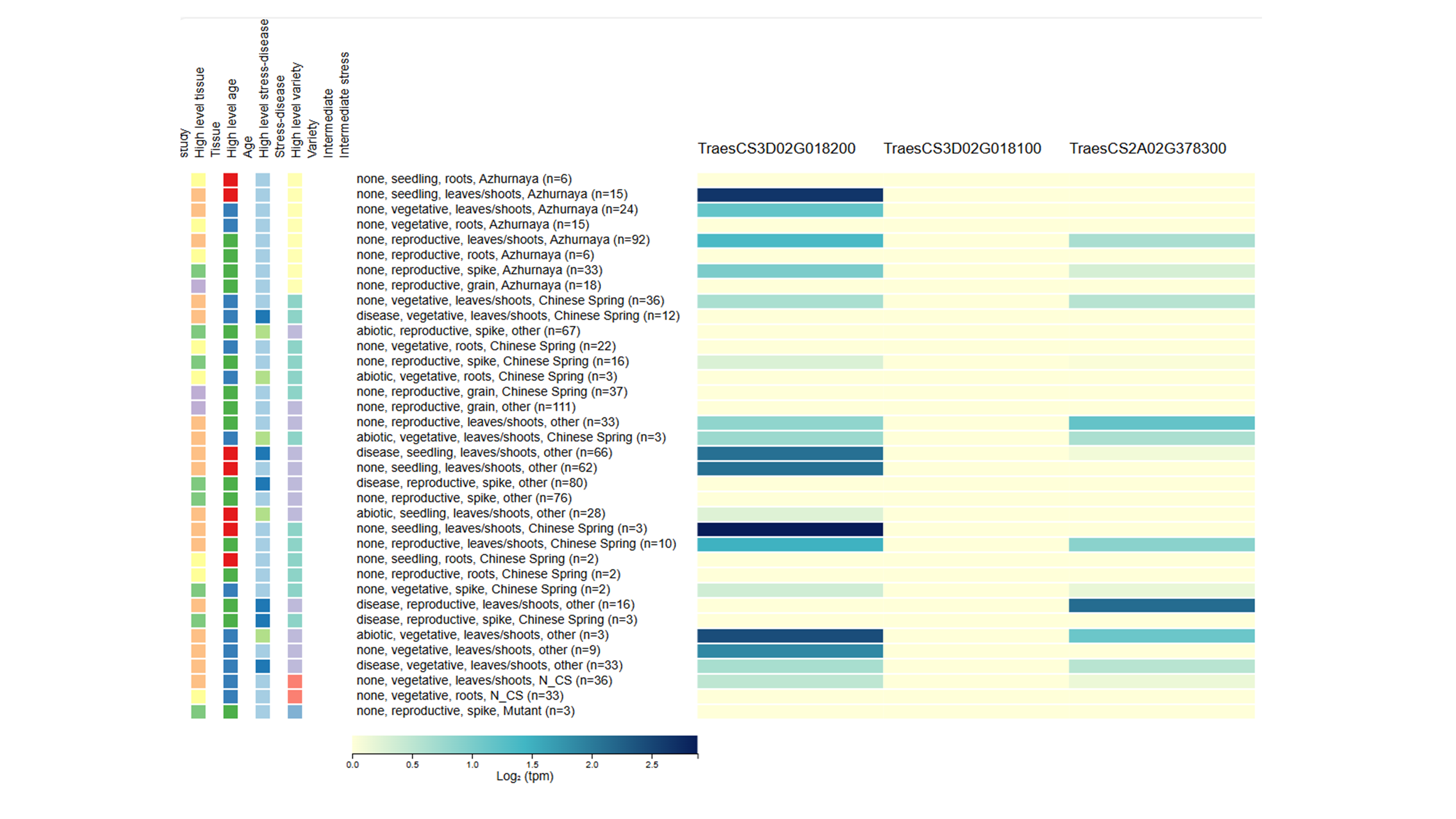

Supplement: S3 Fig — B. In Silico gene expression analysis for soil plant analysis development value (SPAD). C. In Silico gene expression analysis for normalized difference vegetation index (NDVI). D. In Silico gene expression analysis for stem reserve mobilization (SRM). (ZIP) [file pone.0339374.s003.zip › S3B_ Fig.tif]

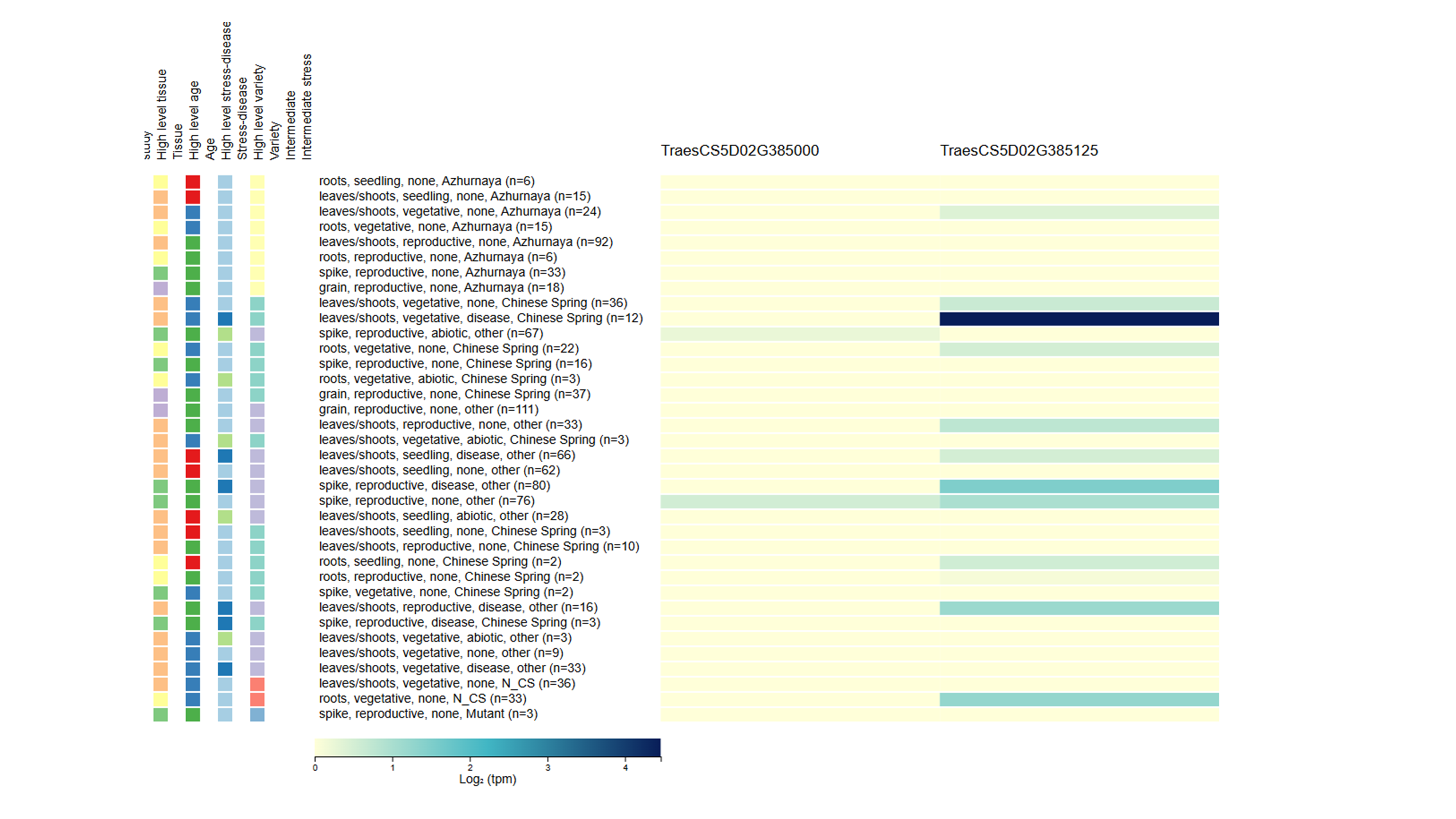

Supplement: S3 Fig — B. In Silico gene expression analysis for soil plant analysis development value (SPAD). C. In Silico gene expression analysis for normalized difference vegetation index (NDVI). D. In Silico gene expression analysis for stem reserve mobilization (SRM). (ZIP) [file pone.0339374.s003.zip › S3C_ Fig.tif]

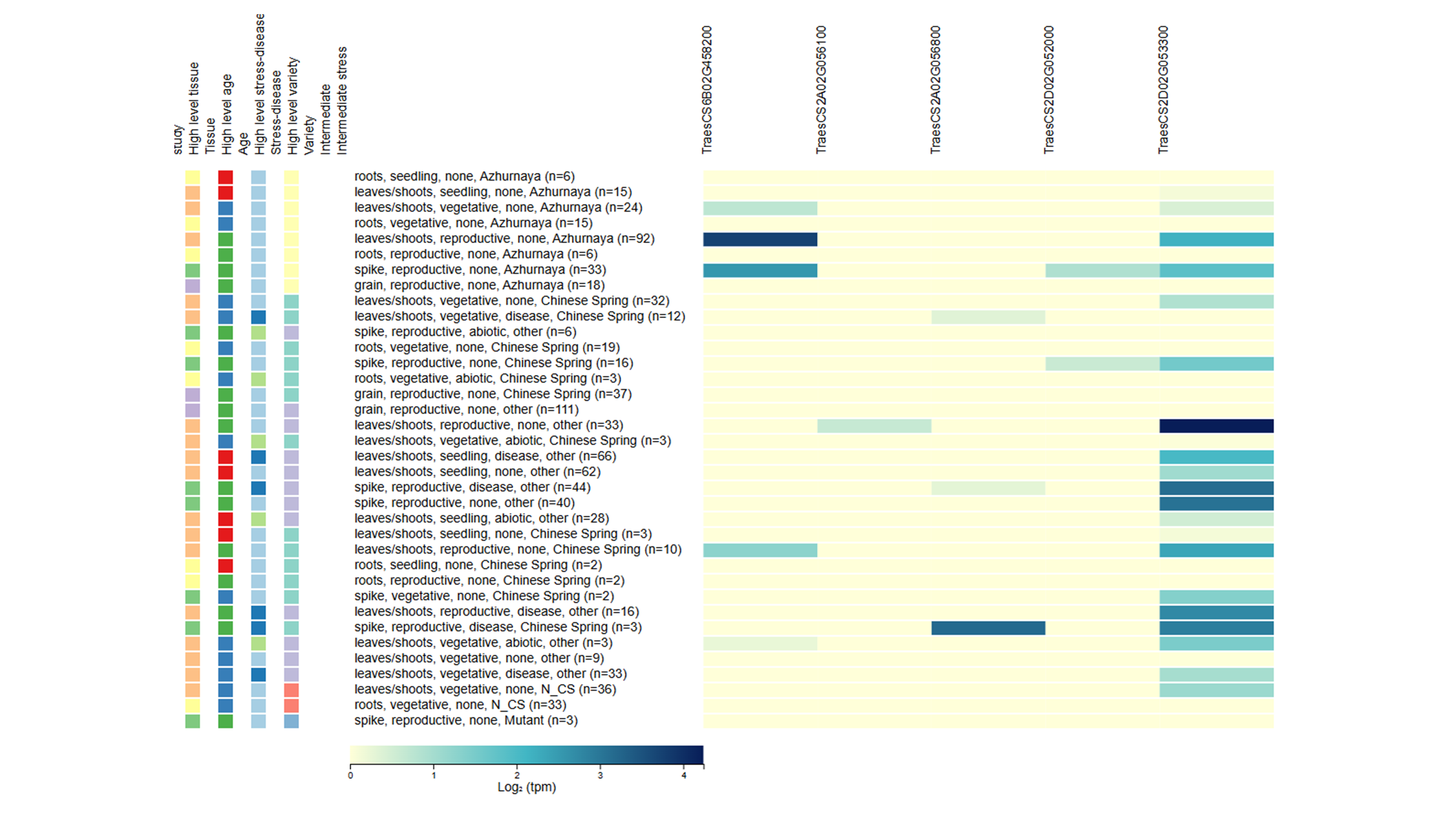

Supplement: S3 Fig — B. In Silico gene expression analysis for soil plant analysis development value (SPAD). C. In Silico gene expression analysis for normalized difference vegetation index (NDVI). D. In Silico gene expression analysis for stem reserve mobilization (SRM). (ZIP) [file pone.0339374.s003.zip › S3D_ Fig.tif]
