# Supplementary material for: Dissecting the differential performance of contrasting stay-green and stem reserve mobilization wheat (Triticum aestivum L.) genotypes – Validation of GWAS analysis
Source: PLoS One. 2026 Jan 5;21(1):e0339374. doi: 10.1371/journal.pone.0339374 (PMC12768281; doi:10.1371/journal.pone.0339374)
Supplement: S1 Table — (DOCX) [file pone.0339374.s004.docx]

| Trait | Gene |  | Primer | Start | Stop | Length | Tm | GC% |
| --- | --- | --- | --- | --- | --- | --- | --- | --- |
| Reference gene | Actin | FP | CCGATCCACACACTGTACTT | 216 | 236 | 20 | 61 | 50 |
|  |  | RP | GACAGGATGAGCAAGGAGAT | 291 | 311 | 20 | 61 | 50 |
| CT | Protein DETOXIFICATION 40-like (*TaPD*) | FP | TTACATCTCGACGGCAACAC | 585 | 605 | 20 | 62 | 50 |
|  |  | RP | GATAATCCACCAGCTCACACTC | 681 | 703 | 22 | 62 | 50 |
|  | Eukaryotic translation initiation factor 3 subunit M-like (*TaEIF3*) | FP | CTCAGAGGCTCGAATCCTATTT | 833 | 855 | 22 | 62 | 45.5 |
|  |  | RP | CCTCATGAACCAGTCCATATCC | 889 | 911 | 22 | 62 | 50 |
|  | probable protein phosphatase 2C (*TaPP2C*) | FP | ACCAAGAGGCTGTTGACATAG | 860 | 881 | 21 | 62 | 47.6 |
|  |  | RP | GTCCTGGATGTTGGGTACTT | 952 | 972 | 20 | 61 | 50 |
|  | F-box protein (*TaFBP*) | FP | ATGACATGCTAGGAGCCATAC | 878 | 899 | 21 | 62 | 47.6 |
|  |  | RP | CCCACACAGAGAGTTCGTAAT | 962 | 983 | 21 | 62 | 47.6 |
|  | Chloroplast stem-loop binding protein (*TaCSL*) | FP | TCTCAGCCCAGAATAGGAGAA | 83 | 104 | 21 | 62 | 47.6 |
|  |  | RP | GGAGAGGAAGAGACCAATGAAC | 186 | 208 | 22 | 62 | 50 |
| SPAD | Putative E3 ubiquitin-protein ligase SINA-like 9 (*TaE3-ubiquitin*) | FP | TCGTGAGTGACCCGATAGAA | 608 | 628 | 20 | 62 | 50 |
|  |  | RP | GGACACAGAGAGAATGCCAAT | 667 | 688 | 21 | 62 | 47.6 |
|  | Putative pentatricopeptide repeat-containing protein (*TaPPR*) | FP | CCATGCGGTTGTTTGAAGAG | 1091 | 1111 | 20 | 62 | 50 |
|  |  | RP | CCTTTGCTGAGTCCATGATTTG | 1162 | 1184 | 22 | 62 | 45.5 |
|  | Cytokinin dehydrogenase 11-like (*TaCKX11*) | FP | GAGGAGGTGTTCTACACAGTTG | 1384 | 1406 | 22 | 62 | 50 |
|  |  | RP | GTAAGATCTCGTCGTTCTGCTC | 1453 | 1475 | 22 | 62 | 50 |
| NDVI | Single MYB histone 5-like (*TaMYB*) | FP | CAAGGTATGGAGTGGGAAGTTG | 62 | 84 | 22 | 63 | 50 |
|  |  | RP | GCGCCACTTGTCCTTTAGAT | 143 | 163 | 20 | 62 | 50 |
|  | Cationic amino acid transporter 1-like (*TaCAAT-like*) | FP | GTCTTCTGCTACACCGAGTT | 370 | 390 | 20 | 61 | 50 |
|  |  | RP | AGTACTCCAGCAGGATGTTG | 474 | 494 | 20 | 61 | 50 |
| SRM | UDP-glucosyltransferase (*TaUGT*) | FP | TCTCCTCTGTTGTGCTTGTATC | 851 | 873 | 22 | 62 | 45.5 |
|  |  | RP | ATCTAACAACCCAGAGGAAAGG | 946 | 968 | 22 | 62 | 45.5 |
|  | ABC transporter C family member 4-like (*TaABC*) | FP | ATTTGGTACAGGAACCGCTATC | 1747 | 1769 | 22 | 62 | 45.5 |
|  |  | RP | CACCTACAACGGTCTCAGTAAAG | 1830 | 1853 | 23 | 62 | 47.8 |
|  | Serine/threonine-protein kinase *(TaSK2*) | FP | GGGTCTCAATGGGTGATTTCT | 749 | 770 | 21 | 62 | 47.6 |
|  |  | RP | CCATACACTTCTGGCCTTTCT | 829 | 850 | 21 | 62 | 47.6 |
|  | Wall-associated receptor kinase 4-like (*TaWAK*) | FP | CACTATTTCTCCGAGGCTCTTT | 219 | 241 | 22 | 62 | 45.5 |
|  |  | RP | TGGTGACGTTGAAGTGGATAG | 305 | 326 | 21 | 62 | 47.6 |
